# The Dental Curriculum of Sweden

**Published:** 1903-02-15

**Authors:** L. Lindström

**Affiliations:** Stockholm


### THE DENTAL CURRICULUM OF SWEDEN.

BY PROFESSOR L. LINDSTRÖM, OF STOCKHOLM.

When the Royal Caroline Institute was intrusted by our government with the organization of dental education in this country, namely, the theoretical and practical studies necessary for the education of the dentists, we had to solve to the best of our abilities exactly the same questions as are submitted to our discussion by the International Commission of Education.

The first of these questions is framed as follows: "What should be the preliminary requirements for admission into dental schools?" There is only one answer to this question, viz: that if dentistry is accepted as a true specialty of the healing art, the dental candidate should be required to posses the same preliminary education as that required of the medical student. The answer to the third and last question, which reads, "What part of the studies that are taught in medical schools should be pursued by the dental student?" must be in harmony with the opinion expressed with regard to the first question. It is very plain that the same series of subjects should be studied by the dentist and by the physician, but as the study of medicine in this country requires from nine to ten years, the greater part of which is devoted to the theoretical branches, and as the commission intrusted with the organization of dental education had been asked to limit the length of the course to three years, it became impossible to arrange a plan of studies based upon a common education for both the dentist and the physician.

Following these principles, we organized our dental education five years ago. The curriculum is so arranged that the course is completed in three years. The curriculum embraces a theoretical course during the first year and a practical one during the second and third. Last year some preliminary work on prosthetic technics was introduced in the first year with very satisfactory results. The theoretical course is given by the professors of the medical school, and the practical course is given in a dental clinic especially organized for this purpose and in laboratories of operative dentistry and dental prosthesis, by teachers attached to these institutions.

The theoretical course embraces the following subjects: (1) Chemistry, Metallurgy and Materia Medica; (2) Anatomy, Histology and Embryology; (3) Physiology and Medical Physics; (4) General Pathology and Bacteriology.

The subjects of the practical course are as follows: (1) Surgery; (2) Dental Surgery; (3) Obturations; (4) Dental Prosthesis and Orthopedia.

I will now endeavor to answer the other questions proposed by the commission of Education:

*Ans.* The first and second semesters are almost entirely devoted to the study of anatomy. The course is divided into two parts, dissection and microscopy. The program comprises general anatomy, systemic and topographic anatomy of the head and neck, special and comparative anatomy of the teeth and mouth; a general survey of the central nervous system and of the organization of the viscera. The course in histology includes general methods of technique, histology of the cells and tissues and special histology of the teeth and neighboring structures. In the course in embryology, a student takes up at the same time the general development of the organic system and the special development of the skull, face and teeth. I attach a special degree of importance to the evolution of the maxillæ and the teeth after birth, during the period of development, and during senile atrophy. The study of chemistry should comprise the most important features of inorganic and organic chemistry, especially in their relation to dentistry—metallurgy being included in this branch. The study of physiology refers to those parts which have a direct bearing upon the functions of the oral tissues. Materia Medica is taken up in so far as it is related to dentistry. Toxicology, antiseptics, and general and local anesthetics are also included in this group. The course in physiology should comprise the most important points of medical physics and human physiology, especially the physiology of the buccal cavity and neighboring structures. The course in general pathology and bacteriology comprises the most important elements of general pathology and pathological anatomy, the more important phenomena of local changes in the circulation, and passive and active changes of tissues, and must be accompanied by demonstrations by means of anatomical preparations. The course also deals with the most important points in the science of micro-organisms and special mycology. After having past the examinations in the subjects here enumerated, the students follow a course in surgery in the hospital during their second year. This course comprises elements of general surgical pathology, diseases of the buccal cavity and neighboring structures, including diseases of the skin and syphilis and aberrations of development. In Sweden, the students, as a rule, take up the study of dentistry at the age of nineteen or twenty, and after studying three or four years, take the professional examination which confers upon them the right to practice. They enter upon the practice of their profession at an age where we may properly presume that they have a serious conception of their duties as practitioners of dentistry.—*Dental Cosmos.*

